# Electrophysiological study as a predictor of mortality in unexplained syncope

**DOI:** 10.1002/joa3.12836

**Published:** 2023-03-06

**Authors:** Javier Pinos, Gustavo Glotz De Lima, Roberto Sant'Anna, Marcelo Lapa Kruse, Marco Antônio Vinciprova Dall'Agnese, Pedro Henrique Torres Tietz, Marco Aurélio Lumertz Saffi, Tiago Luiz Luz Leiria

**Affiliations:** ^1^ Cardiology Institute of Rio Grande Do Sul University Foundation of Cardiology Porto Alegre Brazil; ^2^ Hospital del Rio Cuenca Ecuador; ^3^ Universidad de Cuenca Cuenca Ecuador; ^4^ Federal University of Health Sciences of Porto Alegre Porto Alegre Brazil; ^5^ Hospital de Clínicas de Porto Alegre Porto Alegre Brazil

**Keywords:** electrophysiological study, prognosis, split his, syncope, ventricular tachycardia

## Abstract

**Background:**

Electrophysiological study can help in the diagnosis of arrhythmic syncope. According to the electrophysiological study finding, the prognosis of patients with syncope is still a matter of study.

**Objective:**

The aim of this study was to assess the survival of patients undergoing electrophysiological study according to their findings and to identify clinical and electrophysiological independent predictors of all‐cause mortality.

**Methods:**

A retrospective cohort study included patients with syncope who underwent electrophysiological study from 2009 to 2018. A Cox logistic regression analysis was performed to identify independent prognostic factors for all‐cause mortality.

**Results:**

We included 383 patients in our study. During a mean follow‐up of 59 months, 84 (21.9%) patients died. The split His group had the worst survival compared with the control group, followed by sustained ventricular tachycardia and HV interval ≥ 70 ms, respectively (*p* = .001; *p* < .001; *p* = .03). The supraventricular tachycardia group showed no differences compared with the control group (*p* = .87). In the multivariate analysis, independent predictors of all‐cause mortality were Age (OR 1.06; 1.03–1.07; *p* < .001); congestive heart failure (OR 1.82; 1.05–3.15; *p* = .033); split His (OR 3.7; 1.27–10.80; *p* = .016); and sustained ventricular tachycardia (OR 1.84; 1.02–3.32; *p* = .04).

**Conclusion:**

Split His, sustained ventricular tachycardia, and HV interval ≥ 70 ms groups had worse survivals when compared to the control group. Age, congestive heart failure, split His, and sustained ventricular tachycardia were independent predictors for all‐cause mortality.

## Graphical abstract

Our analyses suggested that survival free of all‐cause mortality is different according to the electrophysiological study (EPS) findings.

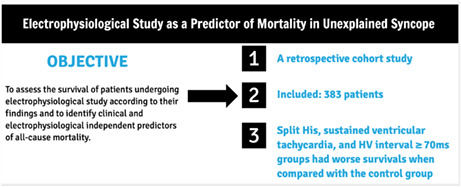

## 1 INTRODUCTION

Patients hospitalized for syncope are at high risk of mortality and cardiovascular events.^1^ However, the prognosis differs depending on the cause of syncope.^2^ High‐risk patients are commonly those with syncope of cardiac origin. Structural heart disease and primary electrical diseases are the main risk factors for sudden cardiac death (SCD) and total mortality in patients with syncope.^3^, ^4^, ^5^

Current clinical guidelines recommend an electrophysiological study (EPS) when an arrhythmic cause of syncope is suspected.^6^, ^7^ There are currently three indications for performing EPS in a context of unexplained syncope: Patients with asymptomatic sinus bradycardia when sinus pauses are suspected, and patients with the bi‐fascicular block on the electrocardiogram (ECG) and suspected tachyarrhythmia as the cause of syncope.^6^ Specifically, the parameters to be evaluated in the EPS are corrected sinus node recovery time (cSNRT), atrioventricular (AV) conduction intervals, especially the HV interval and the presence of intra‐ or infra‐Hisian conduction disturbances, and induction of supraventricular tachycardias (SVT) or ventricular tachycardias (VT).^7^ However, the prognosis of patients seems to vary according to the EPS findings. Currently, there are few data on the prognosis of patients with syncope according to the EPS finding and the predictors of mortality in this group of patients. This study aimed to assess the survival of patients undergoing EPS according to their findings and to identify clinical and electrophysiological independent predictors of all‐cause death.

## 2 METHODS

This is a retrospective, observational cohort study, which included patients with syncope of probable arrhythmic cause and who underwent an EPS. This study was approved by the *IC‐FUC* research ethics committee under case number 5772/19. All data verified in the profile studied followed the premises of the Declaration of Helsinki and the Nuremberg Code, respecting the Research Standards Involving Human Beings (Resolution No. 466/2012), of the National Health Council.

### 2.1 Inclusion criteria

All adult patients, who, after undergoing noninvasive tests, were diagnosed with unexplained syncope, in whom a probable arrhythmic origin was presumed, and who underwent EPS in the period from January 1, 2009, to December 31, 2018, were included in the study. Medical history, physical examination, ECG, laboratory tests, and subsequent cardiology and electrophysiology follow‐up data after EPS were obtained from physical and electronic records. The EPS data were obtained from the electrophysiological records and the EPS reports corresponding to each patient.

### 2.2 Exclusion criteria

Patients under 18 years of age, severe aortic valve stenosis, obstructive hypertrophic cardiomyopathy, or another structural heart disease that could explain syncope without the need for EPS were excluded; in addition to channelopathies such as Brugada syndrome, long QT syndrome, short QT syndrome, early Repolarization syndrome, and catecholaminergic polymorphic ventricular tachycardia. Patients with neurally mediated syncope or in whom a cause was detected by noninvasive tests were also excluded.

### 2.3 Primary outcome

Evaluate the EPS as a prognostic tool in patients with arrhythmic syncope, given by the survival of patients according to their findings in the EPS.

### 2.4 Secondary outcome

Identify clinical and electrophysiological independent predictors of all‐cause mortality in patients with syncope of arrhythmic cause.

#### 2.4.1 Clinical data

Age, gender, diseases such as arterial hypertension, type II diabetes, ischemic heart disease (IHD), congestive heart failure (CHF), and structural heart disease were collected from the medical history. Left ventricular ejection fraction (LVEF) was obtained from echocardiogram data. ECG characteristics were also collected, specifically conduction disturbances.

#### 2.4.2 EPS

The procedures were performed in the Electrophysiology Laboratory of our institution with a “C” Arc fluoroscopy device. A Prucka® workstation and a Medtronic stimulator were used during the procedures. All patients underwent intravenous sedation. The right femoral veins were punctured with the introduction of two sheaths. Fluoroscopy with traditional technology was used as a reference for introducing and placing two quadripolar catheters in their specific locations, initially in the right atrium and His bundle region. Later, the right atrium catheter was moved to the right ventricle for the ventricular stimulation protocol. In the case of a history of palpitations, one of the catheters used was a decapolar catheter placed inside the coronary sinus. cSNRT was obtained after 30–60 s of atrial stimulation with cycles of 600 and 400 ms, and the highest value was corrected for basal heart rate. EPS was considered positive according to current syncope guidelines^6^, ^7^ in the following cases: cSNRT > 525 ms, baseline HV interval ≥ 70 ms, or ≥100 ms after 1C type drug administration, second, or third‐degree infra‐His block during incremental atrial stimulation or after administration of 1C antiarrhythmic drugs, and intra‐Hisian conduction disturbance (split His). Patients with any of these findings received a pacemaker (PM). The programmed ventricular stimulation protocol was performed in all patients with structural heart disease with up to three extra stimuli and a shorter coupling interval of 200 ms. Patients who induced sustained monomorphic VT were considered positive for an implantable cardioverter defibrillator (ICD). Patients who induced polymorphic ventricular tachycardia/ventricular fibrillation (PVT/VF) were considered positive for an ICD when they were induced with one or two ventricular extra stimuli and with a shorter coupling interval of 200 ms; in addition, the induced arrhythmia had to be sustained and in need of defibrillation.^8^, ^9^

### 2.5 Definition of variables

All‐cause mortality: Defined as mortality from any cause. It was obtained from the Civil Registry of the State of *“Rio Grande do Sul”* and confirmed through calls to the telephone numbers registered in the hospital's database.

Ischemic heart disease: Defined as coronary artery obstruction diagnosed by any currently recommended imaging method, history of acute coronary syndrome with a demonstration of coronary lesions, previous myocardial infarction, or a history of coronary angioplasty.

Congestive heart failure: Defined as a structural or functional abnormality that results in elevated intracardiac pressures and/or inadequate cardiac output with at least one episode of fluid retention or pulmonary congestion that required medical treatment.

Structural heart disease: Defined as a previous diagnosis of ischemic heart disease with structural cardiac remodeling seen by imaging tests, heart failure, valve dysfunction (mild valve regurgitation was not included in this group) except for severe aortic valve stenosis or primary myocardial structural disease.

**FIGURE 1:**
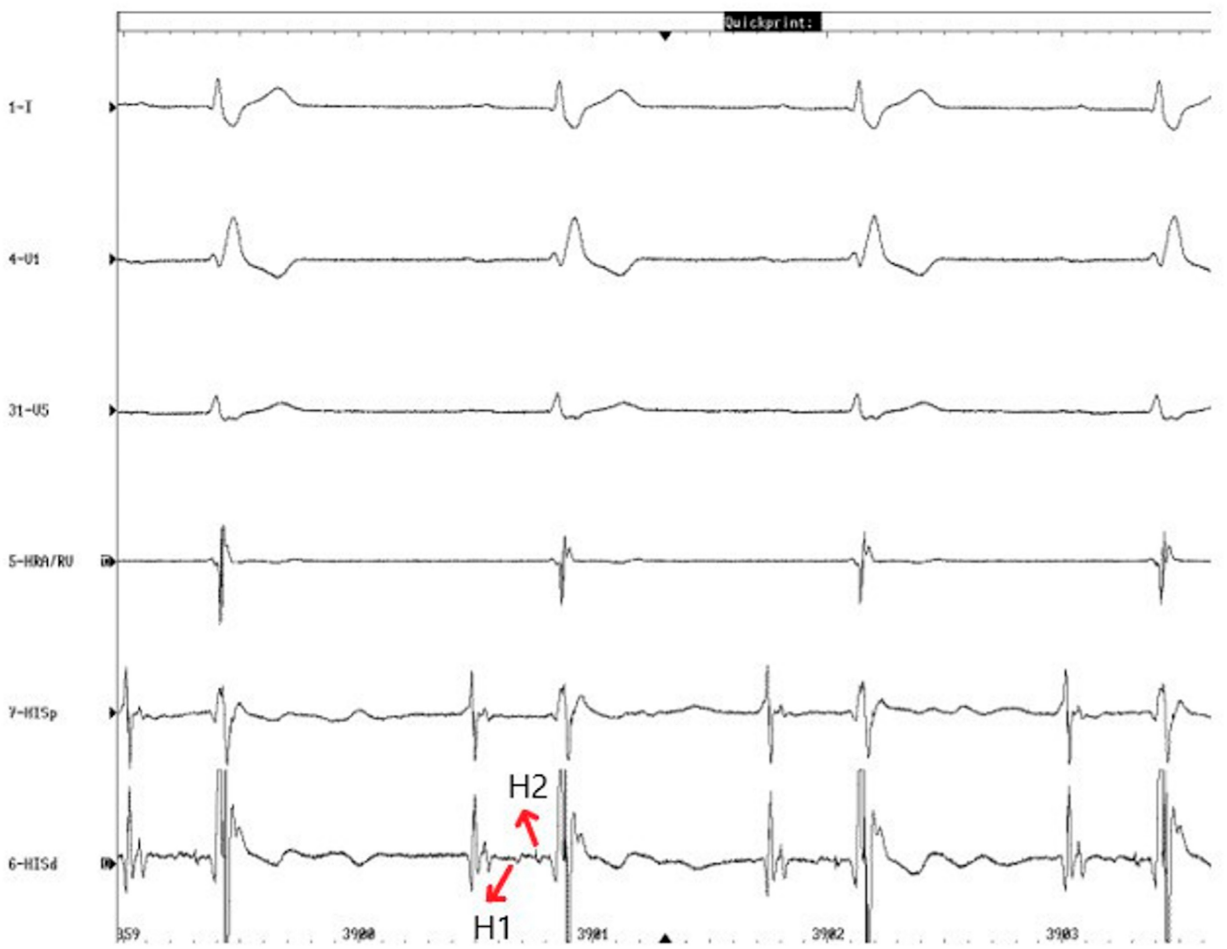
Patient with split His and its two components, “H1” and “H2”.

Split His: Recording two components in the His electrogram (H1 and H2) characterizing fragmentation of the His potential with a duration longer than 25–30 ms^10^ (Figure 1).

### 2.6 Follow‐up

All information was obtained from physical or electronic medical records regarding medical history, physical examination, laboratory tests (including ECG and echocardiogram), procedures, outpatient care, emergency care, and in‐hospital death records. Clinical follow‐up was carried out until December 31, 2020. All patients (or family) were contacted by telephone in January 2021 to obtain information about the occurrence of death outside the hospital. Patients were excluded from the study if it was impossible to establish any form of contact to determine their status at the end of the follow‐up period (seven patients). This study was carried out according to the *Strobe statement*.^11^

### 2.7 Statistical analysis

The database was generated using SPSS Mac OS 25.0 software version (IBM SPSS Statistics). Continuous variables are described as mean ± SD or median with a 95% confidence interval for that value. Categorical variables are represented as absolute numbers and percentages. A descriptive analysis of clinical, ECG, and EPS‐related variables was performed. Survival free from all‐cause mortality was assessed using the Kaplan‐Meier method. The level of significance was defined as *p* < .05. Univariate analysis was performed using the chi‐squared test, Fisher's exact test, or the *t‐test*, as needed. Variables with *p* < .05 were included for multivariate analysis. A multivariate Cox proportional hazard model was used to analyze the variables related to all‐cause death. The study was registered and approved for completion by our institution's research ethics committee, according to Helsinki's declaration.

## 3 RESULTS

**FIGURE 2:**
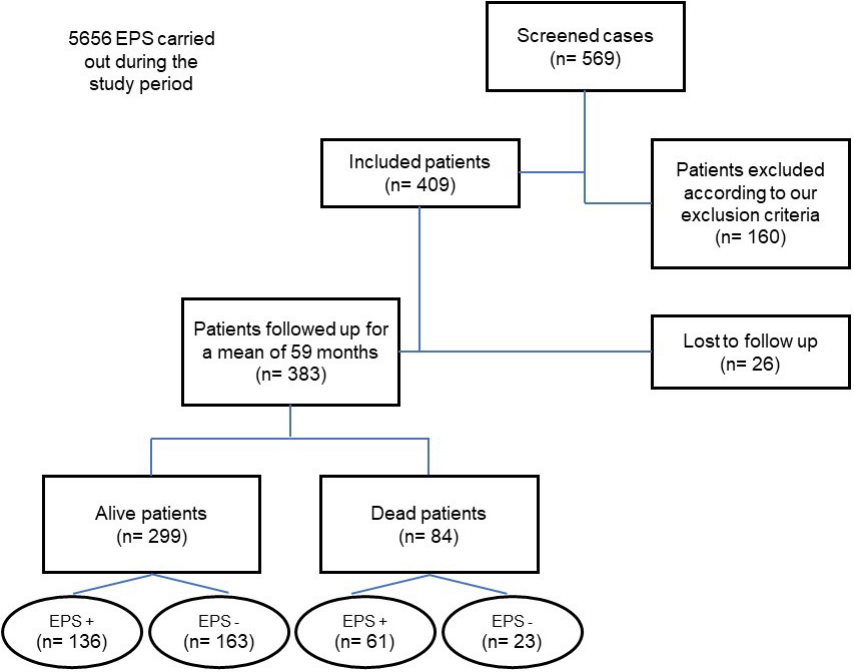
Flowchart representing the study design. EPS, electrophysiology study; +, positive; −, negative.

During the study period, 5656 EPS were performed in our laboratory. Of these, 569 were in the assessment of unexplained syncope; 409 patients met the inclusion and exclusion criteria described. During follow‐up, 26 were excluded due to a lack of data. The remaining 383 patients (Figure 2) were included, and their clinical characteristics are shown in Table 1.

**TABLE 1:** Baseline characteristics.

Baseline characteristics	*N* = 383
Age (years)	64 ± 15
Male gender	245 (64)
Hypertension	259 (67.6)
Diabetes	79 (20.6)
Ischemic heart disease	138 (36)
Heart failure	67 (17.5)
Structural heart disease	181 (47.3)
Left ventricular ejection fraction (LVEF) (%)	49.3 ± 15.7
Multiple episodes of syncope	164 (42.8)
Pacemaker/Cardiac resynchronization therapy	144 (37.6)
Implantable cardioverter defibrillator	53 (13.8)
Electrocardiogram characteristics	
First‐degree atrioventricular block	39 (10.2)
Left bundle branch block	48 (12.5)
Right bundle branch block	9 (2.3)
Left anterior or posterior fascicular block	17 (4.4)
Bi‐fascicular block	24 (6.3)
First‐degree atrioventricular block and Bi‐fascicular block	11 (2.8)

*Note*: Data are expressed as mean ± standard deviation or number (%).

The mean age of participants was 64 ± 15 years, and 245 (64%) were men. Comorbidities included arterial hypertension (259; 67.6%), diabetes mellitus (79; 20.6%), IHD (138; 36%), CHF (67; 17.5%), and structural heart disease (181; 47.3%), and 197 patients (51.4%) had implanted a cardiac implantable electronic device (CIED) (70; 18.3% was ICD). In our study, first‐degree atrioventricular block (AVB) was present in 39 patients (10.2%). The distribution of the ECG patterns was left bundle branch block (LBBB) in 48 (12.5%), right bundle branch block (RBBB) in 9 (2.3%), left anterior fascicular block/left posterior fascicular block (LAFB/LPFB) in 17 (4.4%), bi‐fascicular block in 24 (6.3%), and first‐degree AVB plus bi‐fascicular block in 11 (2.8%). The EPS was positive in 197 (51.4%) patients, and more than one pathological finding was evidenced in some cases. The mean procedure duration was 43 min; the mean fluoroscopy time was 3.8 min. No serious complication was recorded. A total of 38 supraventricular tachycardia ablations were performed. A cSNRT > 525 ms was present in 58 (15.1%) patients, an HV interval ≥ 70 ms was found in 87 (23.2%) patients, split His was evidenced in 8 (2.1%) patients, and sustained VT was induced in 70 (18.3%) patients (Table 2).

**TABLE 2:** Electrophysiological study results.

Parameter	*N* = 383
Positive EPS	197 (51.4)
SNRT > 1500/CSNRT > 525 (ms)	65 (17)
HV ≥ 70 (ms)	87 (23.2)
Split His	8 (2.1)
Induction of nonsustained MVT	11 (2.9)
Induction of sustained MVT	35 (9.1)
Induction of nonsustained PVT/VF	6 (1.6)
Induction of sustained PVT/VF	18 (4.7)

*Note*: Data are expressed as number (%).

Abbreviations: cSNRT: corrected sinus node recovery time; EPS: electrophysiology study; MVT: monomorphic ventricular tachycardia; PVT: polymorphic ventricular tachycardia; VF: ventricular fibrillation.

Patients with cSNRT >525 ms, HV interval ≥ 70 ms, or ≥100 ms after 1C type drug administration, second, or third‐degree infra‐His block during incremental atrial stimulation or after 1C antiarrhythmic drugs administration, and intra‐Hisian conduction disturbance (split His), received a PM implant. Patients who induced sustained VT received an ICD implant in all cases.

**FIGURE 3:**
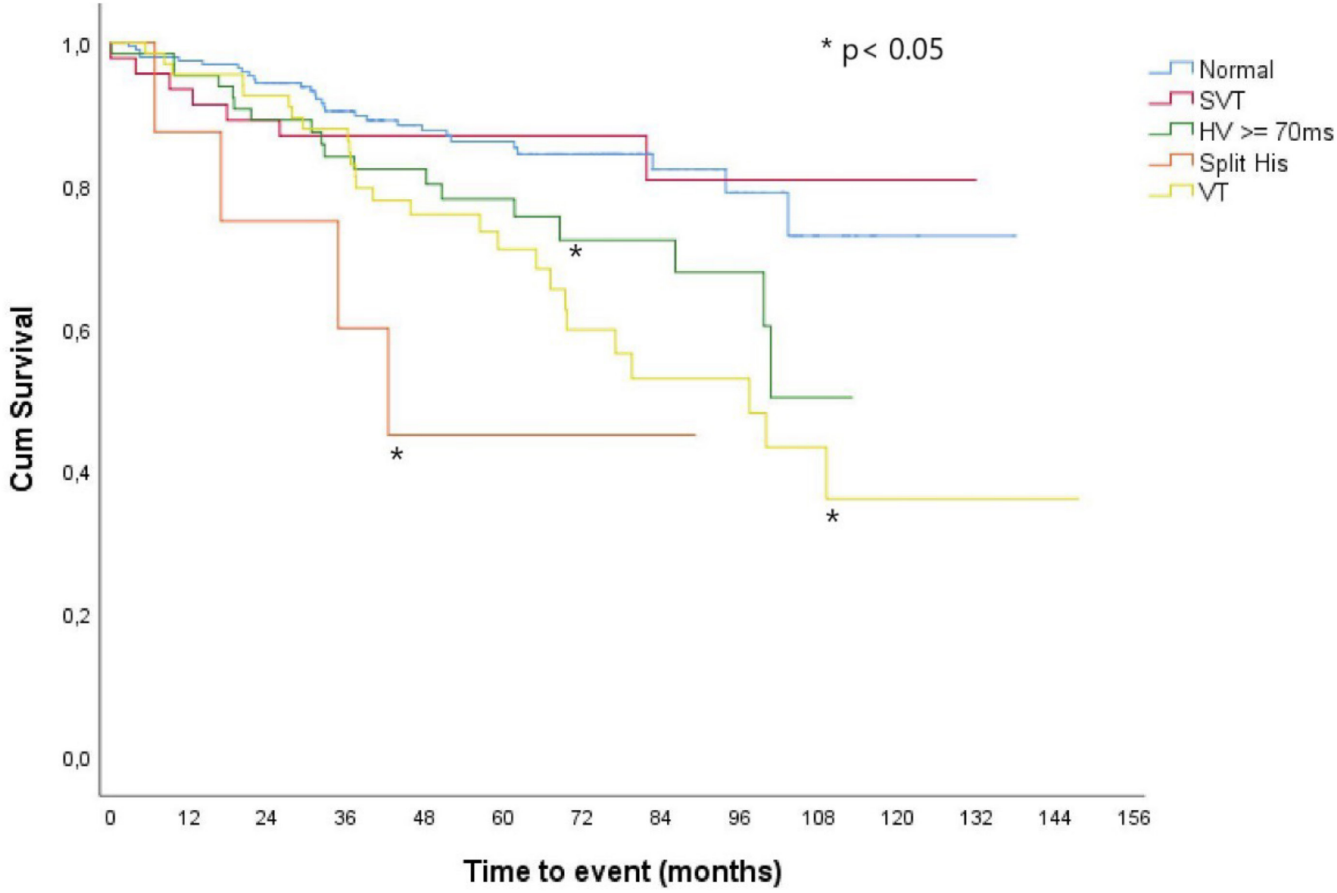
Comparison of mortality‐free survival curves between the four groups studied and the control group. SVT, supraventricular tachycardia; VT, ventricular tachycardia.

During a mean follow‐up of 59 months, 84 (21.9%) patients died (Table 3). Survival was analyzed according to four groups (SVT, HV ≥70 ms, split His, and sustained VT) and compared with the group without these findings (control group). Three groups showed differences when compared to the control group: split His, VT, and HV ≥70 ms (*p* = .001; *p* ≤ .001, *p* = .03, respectively). The SVT group showed no differences compared with the control group (*p* = .87) (Figure 3). Of the analyzed variables, the univariate predictors for total mortality were age, CHF, IHD, structural heart disease, HV ≥70 ms, split His, and sustained VT (*p* ≤ .05) (Table 4). The multivariate model included these variables and was analyzed by multivariate logistic regression. In multivariate analysis, the only independent predictors of all‐cause mortality were Age (OR 1.06; 1.03–1.07; *p ≤* .001); CHF (OR 1.82; 1.05–3.15; *p* = .033); split His (OR 3.7; 1.27–10.80; *p* = .016); and sustained VT (OR 1.84; 1.02–3.32; *p* = .04) (Table 5).

**TABLE 3:** Comparison of clinical and electrophysiological characteristics between patients who died and those who survived (*N* = 383).

Patient characteristics	Death *N* = 84	Survival *N* = 299	*p* value
Sinus rhythm	71 (84.5)	284 (94.9)	<.05
Baseline atrial fibrillation rhythm	11 (13)	13 (4.3)	<.05
Baseline pacemaker rhythm	2 (2.4)	2 (0.66)	NS
First‐degree AV block	14 (16.6)	25 (8.3)	<.05
Mobitz 1 s‐degree AV block	‐	4 (1.3)	NS
cSNRT > 525 ms	9 (10.7)	49 (16.3)	NS
Supraventricular tachycardia	13 (15.4)	49 (16.3)	NS
HV ≥ 70 ms	61 (72.6)	53 (17.7)	<.05
Split His	4 (4.7)	4 (1.3)	NS
Sustained MVT	17 (20.2)	18 (6)	<.05
Sustained PVT/VF	4 (4.7)	14 (4.7)	NS

*Note*: Data are expressed as number (%). Abbreviations: AV, atrioventricular; cSNRT, corrected sinus node recovery time; MVT, monomorphic ventricular tachycardia; PVT, polymorphic ventricular tachycardia; VF, ventricular fibrillation; NS, not significant.

**TABLE 4:** Univariate analysis of predictors of all‐cause death.

Variables	Univariate	
	OR (95% confidence interval)	*p* value
Age	1.049 (1.029–1.069)	<.001
Congestive heart failure	2.771 (1.767–4.346)	<.001
Structural heart disease	2.416 (1.539–3.793)	<.001
Ischemic heart disease	1.549 (1.002–2.393)	.049
Supraventricular tachycardia	1.060 (0.464–2.423)	.89
HV ≥ 70 (ms)	1.857 (1.031–3.346)	.039
Split His	5.284 (1.850–15.091)	.002
Sustained ventricular tachycardia	2.593 (1.525–4.409)	<.001

Abbreviation: HV, His‐Ventricle interval.

**TABLE 5:** Multivariate analysis of independent predictors of all‐cause death.

Independent predictors of all‐cause mortality	OR	CI 95%	*p* value
Clinical predictors			
Age	1.06	1.03–1.07	<.001
CHF	1.82	1.05–3.15	.033
EPS predictors			
Split His	3.7	1.27–10.80	.016
Sustained VT	1.84	1.02–3.32	.04

Abbreviations: CHF, congestive heart failure; VT, ventricular tachycardia.

## 4 DISCUSSION

Cardiac syncope is not only related to a worse prognosis, but it is also a strong predictor of mortality.^12^ Structural heart disease and primary electrical disease are the main risk factors for SCD and total mortality in this subgroup of patients.^3^, ^4^ The EPS is useful when there is an arrhythmic cause. However, it appears that patients' prognoses differ depending on the arrhythmic finding. This study shows that survival free of all‐cause mortality is different according to the EPS findings.

Of the four groups studied, when compared to the control group, the one with the worst survival was the split His group, followed by the sustained VT and HV interval ≥ 70 ms group, respectively. On the contrary, the SVT group showed no differences compared with the control group.

The presence of split His is an infrequent finding, and little is known about its impact on survival. Lerman et al.^13^ described a case series of patients with intra‐Hisian conduction disorders, where five had split His. Of this group, two cases developed VT at follow‐up, and two patients registered SCD. In another case series of 16 patients (12 with PM implant) described by Gupta et al.,^14^ 13 patients had split His, and 10 deaths were recorded in the follow‐up, showing high mortality in this specific group. This relationship has also been demonstrated in our study, positioning the split His group as the one with the worst prognostic compared with the control group and being the strongest independent predictor of all‐cause mortality (OR 3.7; 1.27–10.80; *p* = .016). These findings were even after PM implantation in all cases. It is important to note that patients who received PM implantation and died during follow‐up did not present PM dysfunctions in the subsequent controls. In the cause of death records of the four patients, one was due to sudden death, and the other three have an “undetermined” cause of death.

The group of patients who developed sustained VT on EPS showed worse survival when compared to the control group. It is known that VT induction in EPS is a marker of poor prognosis, but this finding seems to differ depending on the underlying disease. Brembilla‐Perrot et al.^15^ demonstrated that in IHD, VT or VF induction was a predictor of mortality; on the contrary, in nonischemic dilated cardiomyopathy, only LVEF proved to be an independent predictor. Another study secondarily evaluated the total mortality in patients with IHD and sustained monomorphic VT induction, observing higher mortality in this group compared with the group without sustained monomorphic VT induction.^16^ In our series, the prevalence of CAD was 36%, which probably influenced the finding of sustained VT as an independent predictor of total mortality (OR 1.84; 1.021–3.325; *p* = .04). It should be noted that of the patients with ICD who died during follow‐up, only 5 (16.6%) patients had a history of appropriate ICD shocks; on the contrary, 25 (83.4%) patients did not have a history of shocks. No inappropriate shocks were recorded in this subpopulation.

Currently, the recommendation for PM implantation in patients with syncope is if, in addition to bi‐fascicular block on the ECG, they present an HV interval ≥ 70 ms on the EPS.^16^ However, this strategy reduces the recurrence of syncope but does not change the mortality.^17^, ^18^ In our study, although the group of patients with an HV interval ≥ 70 ms had worse survival than the control group, the multivariate analysis did not identify it as an independent predictor of all‐cause death.

Among the clinical factors studied, only age and CHF were independent predictors of mortality in our study. These two variables have been recognized as predictors of mortality, not only in patients with syncope but also in other cardiovascular scenarios.^12^, ^19^ Another study that evaluated the cause of syncope and predictors of mortality in patients hospitalized for syncope did not find age and CHF as independent predictors of death. However, in that study, the main cause of syncope was an “undetermined cause”, followed by vasovagal syncope, and only a small percentage had tachyarrhythmias and bradyarrhythmias as the cause of syncope. Furthermore, only 8% had a history of CHF.^20^ In our series, 47% had structural heart disease, and 18% had a history of CHF, which could explain the findings in our study.

Implantable loop recorder has proven to be useful in determining the cause of syncope when other techniques, including EPS, have been negative for the diagnosis, especially in patients with conduction disturbances.^21^, ^22^ Randomized studies comparing these two techniques are necessary to determine the best strategy in the evaluation of unexplained syncope.

### 4.1 Limitations

This is an observational, retrospective, and single‐center study and will need to be repeated in a prospective and larger trial to confirm our results. Another limitation is the small number of patients in the split His group, which may overestimate the results, but it is consistent with daily clinical practice and the small number of patients who present this alteration in the EPS. It is a retrospective study where we only have the data on whether the patients died, and we do not know whether the death was because of arrhythmic causes. Another limitation is the fact that patients who have not been contacted at the end of follow‐up were excluded; this could result in survival bias as the primary outcome might have been missed because of that.

## 5 CONCLUSION

We evaluated the survival and all‐cause mortality predictors in patients undergoing EPS because of unexplained syncope in four groups that were compared with the control group. Split His, sustained VT and HV interval ≥ 70 ms groups, had worse survivals when compared to the control group. The SVT group's survival was not different compared with the control group. Independent predictors for all‐cause mortality on EPS were the presence of split His and sustained VT. Independent clinical predictors for all‐cause mortality were age and history of CHF.

## AUTHOR CONTRIBUTIONS

Javier Pinos and Tiago Luiz Luz Leiria contributed to the conception, design, analysis, and interpretation of the data. Marco Antônio Vinciprova Dall'Agnese, Pedro Henrique Torres Tietz, Marco Aurélio Lumertz Saffi, and Tiago Luiz Luz Leiria contributed to the drafting of the article and critical revision. Javier Pinos, Gustavo Glotz De Lima, Roberto Sant'Anna, Marcelo Lapa Kruse, Marco Antônio Vinciprova Dall'Agnese, Pedro Henrique Torres Tietz, Marco Aurélio Lumertz Saffi, and Tiago Luiz Luz Leiria contributed to the final approval of the version to be published. Javier Pinos, Gustavo Glotz De Lima, Roberto Sant'Anna, Marcelo Lapa Kruse, Marco Antônio Vinciprova Dall'Agnese, Pedro Henrique Torres Tietz, Marco Aurélio Lumertz Saffi, and Tiago Luiz Luz Leiria contributed to the agreement to be accountable for all aspects of the work in ensuring that questions related to the accuracy and integrity of the article.

## FUNDING INFORMATION

There were no external sources of funding for this study.

## CONFLICT OF INTEREST STATEMENT

The authors declare that there are no conflicts of interest regarding the publication of this article.
